# ABA Biosynthesis and Signaling Cascades Under Hypoxia Stress

**DOI:** 10.3389/fpls.2021.661228

**Published:** 2021-06-24

**Authors:** Qichao Wang, Lei Wang, Umashankar Chandrasekaran, Xiaofeng Luo, Chuan Zheng, Kai Shu

**Affiliations:** ^1^School of Ecology and Environment, Northwestern Polytechnical University, Xi'an, China; ^2^Shenzhen Research Institute of Northwestern Polytechnic University, Shenzhen, China; ^3^Institute of Ecological Agriculture, Sichuan Agricultural University, Chengdu, China

**Keywords:** ABA, hypoxia, submergence, waterlogging, flooding, ethylene

## Introduction

Hypoxia is one of the major abiotic stresses, primarily caused by numerous flooding events such as waterlogging and submergence (Zhou et al., 2020a; Xie et al., 2021), with deleterious effects on plant growth and development (Bailey-Serres et al., 2012; Voesenek and Bailey-Serres, 2015; Xie et al., 2021; Zhou et al., 2021). Due to the excessive water absorption, hypoxia mechanically damages seed germination, seedling establishment, and finally crop yield (Nakayama et al., 2004; Arguello et al., 2016; Yanjun et al., 2016; Striker and Colmer, 2017; Wang et al., 2017; Shen et al., 2020; Lee et al., 2021; Tian et al., 2021). Further, flooding decreases the seed quality of cotton and soybean by altering the accumulation and distribution of carbohydrates, oil, and protein (Wang et al., 2018; Xu et al., 2021). Collectively, hypoxia stress negatively regulates numerous aspects of plant development.

Abscisic acid (ABA) is an essential phytohormone that regulates plant growth and development, such as seed germination, seed dormancy, seed longevity, and seedling establishment (Zhu, 2016; Khan et al., 2020; Umashankar et al., 2020; Zhou et al., 2020b). It is worth noting that ABA also responds to abiotic stresses such as drought, salt, and water stresses (Zhu, 2016; Shu et al., 2018). ABA induces stomata formation on underwater leaves (Iida et al., 2016) and controls stomatal movement by regulating the size of guard cells, thus mediating water potential in plants (Zhu, 2016; He et al., 2018). During hypoxia stress, ABA biosynthesis is inhibited, while the catabolism cascade is enhanced, and thus, exogenous ABA can increase the tolerance of plants to hypoxia stress (Dawood et al., 2016; De Ollas et al., 2021). Under flooding conditions, pretreatment with ABA increases the abundance of protein through glycolysis, fermentation, and tricarboxylic acid cycle (TCA), thereby enhancing hypoxic properties and improving survival rate in soybean (Komatsu et al., 2013; Yin et al., 2016; Wang et al., 2018). Moreover, the application of ABA positively regulates the net assimilation rate (NAR), relative growth rate (RGR), and chlorophyll content of rice under flooding (Saha et al., 2021). However, these reports did not provide a detailed molecular mechanism of hormone regulation under hypoxia. Therefore, considering the response of ABA to hypoxia stress, we need to understand the detailed molecular mechanisms, especially the underlying mechanisms of ABA biosynthesis, catabolism, and signal transduction under hypoxia stress. Here, this opinion intends to highlight some critical unanswered questions, which need to be addressed in future exploration.

## Abscisic Acid, Ethylene, and Gibberellin (GA) Cross Talk in Response of Plants to Hypoxia Stress

Because of its gaseous nature, it is difficult for ethylene to leave the plant under flooding conditions, so it rapidly accumulates inside the plant and reflects the predicament of plants (Hattori et al., 2009; Alpuerto et al., 2016). Ethylene is the primary signal for adaptation of a plant to flooding (Loreti et al., 2016), which regulates ABA, GA, and auxin, affecting plant growth and development under hypoxia stress (Steffens et al., 2006; Vidoz et al., 2010; Dawood et al., 2016; Yang et al., 2018). Ethylene involves in stimulating bud elongation, aerenchyma development, and adventitious root (AR) formation under flooding conditions (Voesenek et al., 2003; Rajhi et al., 2010; Dawood et al., 2016; Nguyen et al., 2018). As such, ethylene has become a hot topic in hypoxia research like flooding.

**Figure 1:**
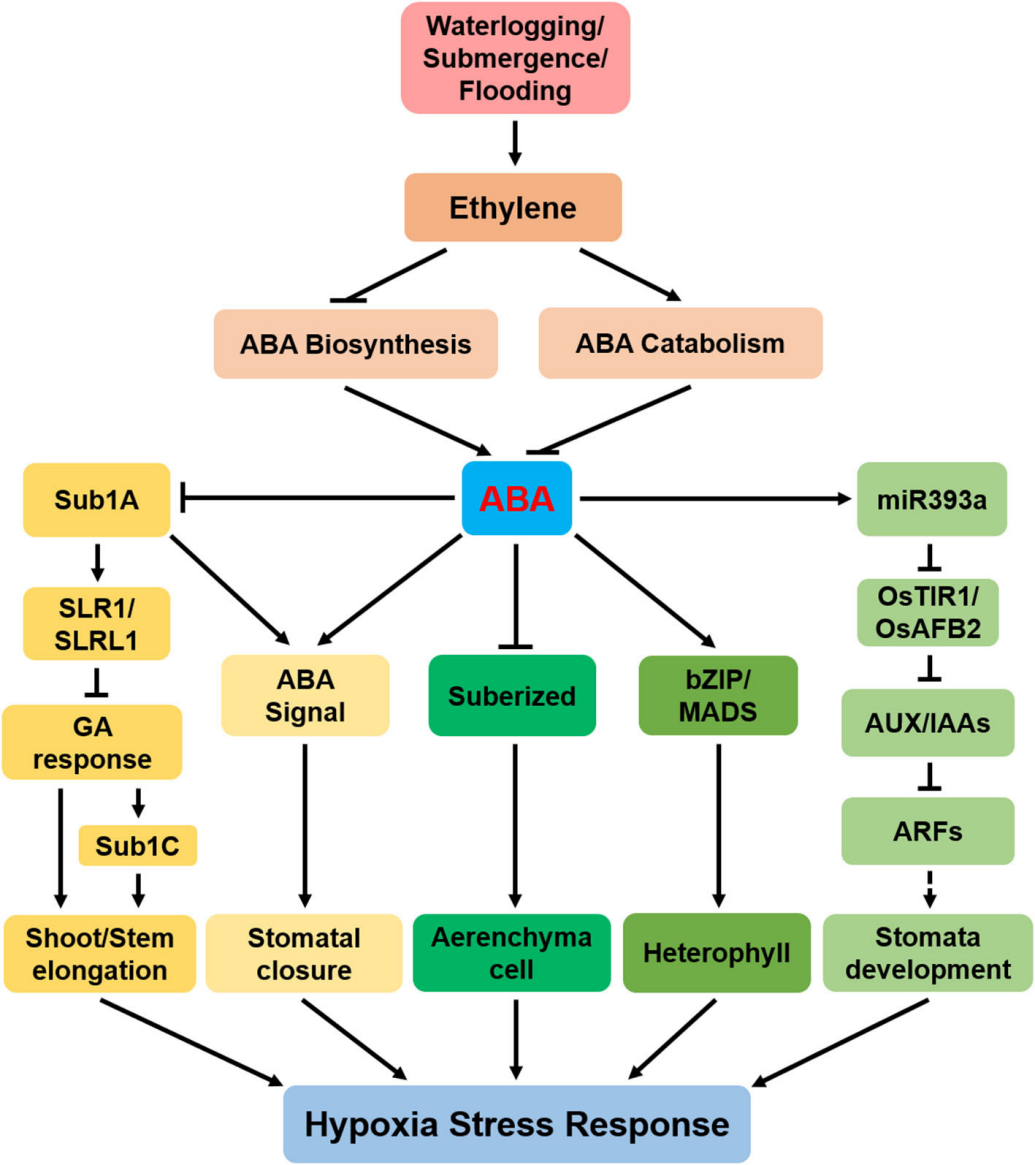
A model showing that abscisic acid (ABA) regulates hypoxia stress response. Submergence, waterlogging, and flooding cause hypoxia, which leads to ethylene accumulation. Ethylene positively regulates ABA catabolism and negatively regulates ABA biosynthesis, thereby affecting ABA content. ABA affects tolerance of plant hypoxia through the following pathways: (1) ABA negatively regulates *submergence1 A* (*Sub1A)* expression. *Sub1A* promotes the accumulation of two gibberellin (GA)-negative regulators [SLENDER RICE 1 (SLR1) and SLENDER RICE-LIKE 1 (SLRL1)] and then directly promotes shoot elongation; (2) ABA signal induces stomata to close; (3) ABA positively regulates the formation of aerenchyma cell by negatively regulating root cell suberization; (4) ABA induces the expression of the basic region-leucine zipper (*bZIP/MADS)* to promote the formation of special-shaped leaves of submerged plants and semiaquatic plants; (5) ABA positively regulates *miR393a*, then negatively regulates auxin signaling cascade, and finally positively regulates stomatal development. The arrows indicate the promotion effect, and the flat lines indicate the inhibition effect. Dotted lines indicate indirect interactions.

Ethylene accumulated under flooding stress induces elongation by inhibiting the biosynthesis of ABA in *Rumex palustris* (Benschop et al., 2005). Thus, with the increased ethylene level under waterlogging conditions, the ABA concentration decreases (Figure 1) and endogenous GA increases. The reduction in ABA is necessary for the submergence-induced GA response, which promotes internode or petiole elongation (Kende et al., 1998; Benschop et al., 2006). Mechanistically, the accumulated ethylene inhibits the expression of 9-cis-epoxycarotenoid dioxygenase encoding genes (*NCEDs)*, which also leads to the breakdown of ABA into phaseic acid (PA), thereby reducing ABA content (Benschop et al., 2005; Saika et al., 2007). A reduction in ABA content interferes with the GA pathway, leading to rapid shoot elongation under submergence, as seen in marsh docks (Benschop et al., 2006) and rice (Kende et al., 1998). Similarly, flooding increases stem elongation in deepwater rice varieties, partially by reducing endogenous ABA content and increasing GA concentration (Yang and Choi, 2006).

Ethylene and its precursor 1-aminocyclopropane-1-carboxylic acid (ACC) induce rapidly the expression of ABA 8′-hydroxylase 1 (*OsABA8ox1*), and pretreatment with the ethylene receptor inhibitor 1-methylcyclopropene (1-MCP) inhibits its expression (Saika et al., 2007). These results suggest that under flooding conditions, the rapid decline of ABA in deepwater rice varieties is partly controlled by the ethylene-induced expression of *OsABA8ox1* (Saika et al., 2007; Pan et al., 2021). Degradation of ABA is enhanced by submergence in *submergence1 A* (*Sub1A*)-independent manner (Figure 1) (Fukao and Bailey-Serres, 2008). At the same time, the exogenous ABA decreases the abundance of the *Sub1* gene, suggesting that during submergence, reduction in ABA content may be a prerequisite for the increased accumulation of *Sub1* transcript (Fukao and Bailey-Serres, 2008). Thus, to determine the cross talk between ethylene and ABA under hypoxia stress, it is necessary to clarify the molecular mechanisms by which ethylene regulates ABA biosynthesis and/or signaling.

## Abscisic Acid Biosynthesis Under Hypoxia Stress

The abscisic acid level in internode meristem and cell elongation zone of submerged plants decreased by 75% in deepwater rice after flooding (Kende, 1992). Similarly, endogenous ABA content in *Rumex palustris* decreases in petioles after submergence (Cox et al., 2004). In addition, a recent study also showed that flooding leads to the decline in ABA level in tomato roots (De Ollas et al., 2021). What is the reason for the decrease in ABA content under hypoxia stress? In rice, the expressions of *OsNCED1, OsNCED2, and OsNCED3* decrease rapidly after submergence (Saika et al., 2007). *AtNCED3* expression is also downregulated in roots under submergence, and endogenous ABA level of root decreases significantly (Hsu et al., 2011). Meanwhile, the upregulation of *AtNCED4* expression in shoots under submergence is also documented (Hsu et al., 2011). These results suggest that regulation of ABA biosynthesis in the aboveground and underground parts is distinct and needs further exploration.

In *Solanum dulcamara*, transcription of two *NCED* genes is significantly downregulated under flooding; thus, ABA content in ARs and stems reduces substantially (Dawood et al., 2016). Further studies found that the partial submersion and complete submersion both have no difference in AR growth, and both of them attenuate the expression of *NCEDs*, thereby reducing ABA content (Yang et al., 2018). With the decrease in expressions of *TaNCEDs* (*TaNCED1* and *TaNCED2*) and ABA content in stem nodes, ARs appeared on stem nodes after waterlogging in *Triticum aestivum* L. (Nguyen et al., 2018). All the available evidence supports the fact that ABA is a negative regulator of AR formation and shoot elongation under hypoxia stress (Figure 1).

## Abscisic Acid Catabolism Under Hypoxia Stress

In plants, the catabolism of ABA has two pathways: One is the direct inactivation to form PA, which is the oxidative inactivation pathway. The other is combined with glucose to produce ABA-glucose ester (ABA-GE) and is named as binding inactivation pathway. In Rumex species and rice, the high expression of *OsABA8ox1* after submergence accelerates the ABA degradation and forms a large amount of PA (Benschop et al., 2005; Saika et al., 2007). Studies have found that ABA-GE is involved in regulating the response of plants to drought, salt, and saline–alkali stresses (Dietz et al., 2000; Xu et al., 2012; Gong et al., 2014; Dong et al., 2015; Wang et al., 2020). However, existing reports have found that ABA-GE level under submergence has not changed in rice (Saika et al., 2007; Fukao and Bailey-Serres, 2008). These analyses confirmed that during submersion, the hydroxylation of ABA to PA is the primary pathway of ABA catabolism.

In deepwater rice, the rapid decrease in ABA content is a prerequisite for the increase in bud elongation (Kende et al., 1998; Steffens et al., 2006). Further research found that transcription of *cytochrome P450 A5* (*CYP707A5*) gene is significantly upregulated under submergence in deepwater rice, thus promoting ABA catabolism (Yang and Choi, 2006). *SdABA8ox* is upregulated in the AR primordia of *Solanum dulcamara* after flooding, and the ABA level gets significantly reduced (Dawood et al., 2016). In *Nasturtium officinale, CYP707A1*, and *CYP707A2* are induced under submergence, showing a decline in ABA level (Müller et al., 2019). Similarly, *CYP707A1-1* expression is induced and the mRNA level of *CYP707A1-2* is downregulated in both partial submersion and complete submersion in *Solanum dulcamara* (Yang et al., 2018). In a nutshell, hypoxia promotes ABA catabolism (Figure 1). However, the specific molecular mechanism of enhanced ABA catabolism under hypoxia needs to be elucidated.

## Abscisic Acid Signaling Under Hypoxia Stress

It has been reported that the expressions of ABA receptor genes *pyrabactin resistance* (*PYR*) and *pyrabactin resistance-like* (*PYL*) increase after flooding (Arbona et al., 2017; De Ollas et al., 2021). This may be feedback for maintaining a certain level of ABA signal under flooded soil. Further, the expressions of *late embryogenesis abundant proteins 5* (*LEA5*), *LEA14-1, LEA14-2*, and *ABA-insensitive 5* (*ABI5*) also reduce under submergence (Yang et al., 2018).

Abscisic acid regulates heterophylly initiation through basic region-leucine zipper (*bZIP*) class genes and *AGAMOUS-like 11* (*AGL11*) gene in *Marsilea quadrifolia* (Hsu et al., 2001; Shan et al., 2009). *OE-SUB1A* enhances the sensitivity to ABA, which is consistent with the inhibition of ABA on seed germination and bud elongation (Fukao et al., 2011). Under waterlogging stress, *RELATED TO APETALA2.6-LIKE* (*RAP2.6L*) inhibits *ABI1* transcription, and the *abi1-1/OE-RAP2.6L* double mutant showed increased sensitivity to ABA, which suggests that the overexpression of *RAP2.6L* modifies the ABA-insensitive phenotype of *abi1-1* mutant (Liu et al., 2012). In particular, the zinc-containing finger/BTB (bric-a-brac, tramtrack, and broad complex) domain-containing protein 47, glycine-rich protein, and rRNA-processing protein Rrp5 associated with ABA response are significantly phosphorylated under flooding stress (Yin and Komatsu, 2015). Meanwhile, ABA inhibits the elongation of the coleoptile by upregulating *miR393a* transcription (Guo et al., 2016). Although there are some studies on the above aspects, the specific mechanism still needs further explanation and clarification.

## Conclusions and Perspectives

The regulation of plant growth and development under waterlogging stress is very complex, and a single hormone might not fully reflect the adaptation strategy of plants to hypoxia. ABA, as the downstream of ethylene, regulates plant response to hypoxia stress (Figure 1).

All the current studies mainly focus on the vegetative growth stage of plants under flooding stress. The following aspects need more attention in future studies: (i) The regulatory mechanism of ABA in seed germination and early seedling morphogenesis under waterlogging stress is a worthy subject. (ii) How does ABA regulate plant reproductive growth under submergence? (iii) Because exogenous ABA can alleviate flooding stress, thus, the development of an anti-waterlogging regulator by modifying ABA is a novel interesting project.

Furthermore, are there unknown genetic factors that control ABA-mediated cascade under waterlogging conditions? Does the kinase involved in the ABA signaling pathway to regulate seed germination, stomatal movement, and reproductive growth in hypoxia environments? These questions are essential to fully understand the hypoxia response of plants and are especially important for crops. Together, a better understanding of ABA biosynthesis and signaling during flooding can further dissect the metabolic and genetic pathways that adapt to flooding pressures and will ultimately help us to develop more resilient crop varieties.

## Author Contributions

QW and KS designed the opinion. LW, XL, and CZ helped in providing the inputs. QW, LW, UC, and KS wrote the manuscript. All authors contributed to the article and approved the submitted version.

## Conflict of Interest

The authors declare that the research was conducted in the absence of any commercial or financial relationships that could be construed as a potential conflict of interest.
